# The role of ADAR1 in human pathophysiology

**DOI:** 10.3389/fcell.2025.1672970

**Published:** 2025-12-18

**Authors:** Jiahao Li, Yushan Xiao, Xiaofeng Li, Yan Dai

**Affiliations:** 1 School of Pharmacy, Southwest Medical University, Luzhou, Sichuan, China; 2 Department of pharmacy, Affiliated Hospital of Southwest Medical University, Luzhou, Sichuan, China

**Keywords:** ADAR1, human diseases, physiological process, immunity, cancer

## Abstract

Adenosine deaminase 1 (ADAR1) is an enzyme acting on double-stranded RNA, primarily responsible for catalyzing the adenosine-inosine deamination reaction of dsRNA.An increasing number of studies have demonstrated that ADAR1 plays a pivotal role in various diseases, including cardiovascular, neurological, and immune disorders, among others. Some of these diseases remain incurable. In addition, ADAR1 is also involved in the development and differentiation of various crucial cells, such as hematopoietic stem cells and nerve cells. This article comprehensively summarizes the regulatory effects of ADAR1 on crucial cells and organs across the immune, nervous, respiratory, blood, and digestive systems, along with its influence on disease progression. The aim is to offer assistance in intervening in cells and treating diseases.

## 1 Introduction

**FIGURE 1:**
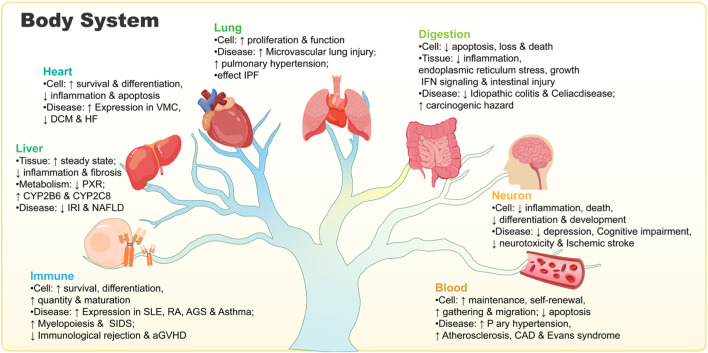
Schematic diagram of the effect of the presence of ADAR1 on various parts of the human body.

ADAR1 acts on RNA, catalyzing the A-I deamination reaction on double-stranded RNA molecules, thereby regulating cellular responses to both endogenous and exogenous RNAs (Sun et al., 2021). ADAR1 is involved in the regulation of the growth and development of cells and organs in the nervous system, respiratory system, digestive system, circulatory system, immune system, endocrine system and the maintenance of homeostasis. Its expression also affects the progression of diseases in various systems. As shown in Figure 1. In addition, we summarize the impact of ADAR1 on various viruses or human diseases. As shown in Table 1.

**TABLE 1:** Overview of the selected ADAR1 gene model.

Model	Phenotype	Cause	Refs
SLE	ADAR1 mRNA content increased	High circulating concentrations of IFN-1 significantly increased ADAR1 mRNA content in T cells	Rönnblom and Alm (2001)
RA	ADAR1 is overexpressed in the synovium of patients	ADAR1 expression decreases when RA treatment is effective	Vlachogiannis et al. (2020)
Bone marrow regeneration	Adar1 promotes infection-induced bone marrow regeneration	ADAR1 binds to and disrupts the stability of Socs3 mRNA in an RNA editing-independent manner	Shen et al. (2018)
Sudden infant death syndrome	ADAR1 is one of the important triggers	ADAR1 induces immune dysregulation	Hafke et al. (2019)
AGS	Mutations in ADAR1	Mutations in K999N, D1113, and p.Gly1007Arg lead to AGS	de Reuver and Maelfait (2024), Inoue et al. (2021), Rice et al. (2012), La Piana et al. (2014), Straussberg et al. (2015), Kono et al. (2016), Garau et al. (2019), Sathishkumar et al. (2021), Liu et al. (2022), Linares-Navarro et al. (2023), Ahmed et al. (2024), Liu et al. (2024), Guo et al. (2021), Guo et al. (2022a), Rice et al. (2017)
Immune rejection	The presence of ADAR1 can inhibit allograft rejection	ADAR1 interacts with miR-21 precursor to promote M2 polarization of mouse splenic macrophages	Ochando et al. (2019), Duneton et al. (2022), Li and Lan (2023), Li et al. (2018)
Liver ischemia-reperfusion injury	ADAR1 alleviates hepatic ischemia-reperfusion injury	ADAR1 increases the expression of anti-inflammatory cytokines and promotes M2 polarization of liver macrophages	Wang et al. (2024)
aGVHD	Overexpression of ADAR1 ameliorates tissue necrosis and reticular formation in liver and lung of aGVHD	Overexpression of ADAR1 decreased the proportion of Th17 and increased the proportion of Treg in aGVHD mice by inhibiting mir-21b	Zhao et al. (2023)
Asthma	ADAR1 expression levels were elevated	Increased expression of type I interferon stimulated genes	Wisnewski and Liu (2024)
IAV	ADAR1 can not only promote IAV protein synthesis and replication, but also activate the innate immune response to limit IAV	ADAR1 promotes virus synthesis and replication by participating in the targeting of NS1, enhances tlr7/8 activation by A-to-I RNA editing of influenza A virus ssRNA, induces innate immune response, and restricts virus	de Chassey et al. (2013), Sarvestani et al. (2014), Vogel et al. (2020), Ward et al. (2011)
HBV	ADAR1 exerts anti HBV effects	ADAR1 inhibits Mavs expression through HuR mediated post transcriptional regulation	Li et al. (2021)
HCV	ADAR1 is involved in clear HCV	ADAR1 participates in the potent antiviral pathway and specifically eliminates HCV RNA through A-to-I RNA editing	Taylor et al. (2005)
Hiv/hcv coinfection	ADAR1 reduces multiple indicators and prevents the occurrence of severe liver disease	Five SNPs in ADAR1 gene (rs1127326, rs1127317, rs1127314, rs1127313, rs2229857) attenuated liver disease	Med et al. (2017)
HDV	ADAR1 inhibits HDV	Both ADAR1-s and ADAR1-l can inhibit HDV replication by editing HDV RNA	Jayan and Casey (2002), Wong and Lazinski (2002), Hartwig et al. (2004)
VSV	ADAR1 enhances VSV replication, and stable knockdown of ADAR1 makes it more responsive to IFN treatment	ADAR1 inhibits PKR activation and enhances VSV replication by a mechanism independent of dsRNA editing	Nie et al. (2007), Li et al. (2010)
LCMV	The expression and activity of adar1-l were significantly upregulated after infection with LCMV	Adar1-l can induce viral RNA mutation leading to loss of viral protein function and reduced viral infectivity	Zahn et al. (2007)
VACV	Activation of ADAR1 helps the virus evade host responses	The E3L gene of vaccinia virus encodes an E3 protein that can activate ADAR1 and compete for Z-type RNA on ADAR1 to suppress innate immune responses	Domingo-Gil et al. (2008), Koehler et al. (2021)
HIV-1	ADAR1 stimulates HIV-1 replication, Knockdown of ADAR1 can inhibit the replication and expression of HIV-1 in a variety of cells *in vivo*	ADAR1 binds to HIV-1 RNA and edits adenosine as well as rev and Tat coding sequences in the 5’untranslated region	Doria et al. (2009), Phuphuakrat et al. (2008), Clerzius et al. (2009)
MeV	ADAR1 restricts MeV replication	ADAR1 can inhibit viral infection by enhancing apoptosis, activating PKR and IRF3, inducing ifn-β RNA, and inhibiting stress granule response. ADAR1 deficient cells show extensive syncytium formation and cytopathic effects	Pfaller et al. (2021), Toth et al. (2009), Li et al. (2012), Okonski and Samuel (2013)
DENV	ADAR1 supports DENV replication	ADAR1 interacts with viral NS3 protein or is regulated by mir-3614-5p to promote viral infection	(de Chassey et al., 2013; Diosa-Toro et al., 2017)
SARS-CoV-2	ADAR1 has the effect of anti-sars-cov-2 and also accelerates the evolution of sars-cov-2 in humans	ADAR1 can mediate the editing of A-to-G in the sars-cov-2 genome	(Gregori et al., 2021; Song et al., 2022)
PyV	Increased virus lethality when ADAR1 is genetically null		George and Samuel (2011)
HTLV	ADAR1 enhances HTLV infection	ADAR1 inhibits HTLV in an edit independent manner and inhibits PKR phosphorylation	Cachat et al. (2014)
HCMV	ADAR1 inhibits HCMV infection	HCMV infection induces the expression of adar1-p110, further upregulates mir-376a and downregulates the immune regulatory molecule HLA-E, making HCMV infected cells easily eliminated	Nachmani et al. (2014)
CVB3	ADAR1 exhibits a double-edged effect upon CVB3 infection	In the early stage of infection, the downregulation of ADAR1 inhibits virus replication and inflammatory response; In the middle and late stages, the downregulation of ADAR1 promotes the inflammatory response through PKR and NF-κB signaling	Dong et al. (2016)
ZIKV	Both ADAR1 knockdown and knockdown significantly reduced ZIKV RNA synthesis, protein levels, and viral titers in cells	ADAR1 inhibits IFN production and PKR activation	Zhou et al. (2019)
ORFV	ADAR1 plays a proviral role	ADAR1 inhibits the phosphorylation of PKR	Liao et al. (2019)
KSHV	Knockdown of ADAR1 inhibits KSHV infection	Knockdown of ADAR1 inhibited viral gene transcription and viral replication during KSHV lytic reactivation and significantly increased type I interferon production during KSHV reactivation	Zhang et al. (2020)
EBOV, MARV	ADAR1 exerts antiviral effects	ADAR1 can edit the 3′UTR in filovirus genome and inactivate mRNA 3′UTR in induced innate immune response	Khadka et al. (2021)
EV-D68	ADAR1 plays a pro viral role	Adar1p110 can directly edit the active site of ev-d68 RNA and its deaminase domain	Zhang et al. (2022b)
MRI	ADAR1 can inhibit apoptosis and inflammation and alleviate injury	The production of ADAR1 is induced to inhibit the activation of PKR during MRI	Wang et al. (2016a)
IRI	Inhibition of ADAR1 significantly enhanced inflammation and liver injury after IRI, while activating IFN response		Wang et al. (2016b)
Nafld	ADAR1 can alleviate nonalcoholic fatty liver disease	Upregulation of ADAR1 inhibits NLRP3 inflammasome activation	Xiang et al. (2022)
Microvascular lung injury	Expression of ADAR1 aggravates the condition of microvascular lung injury	ADAR1 accelerates acute lung injury by mediating the expression of pro-inflammatory and anti-inflammatory cytokines and affecting tissue neutrophil recruitment and D (A-A) O2	(Rabinovici et al., 2001; Wu et al., 2009)
PH	ADAR1 aggravates pulmonary hypertension	ADAR1 participates in m1A modification of circCDK17 and subsequent proliferation of pulmonary artery smooth muscle cells	Zhang et al. (2023)
IPF	Overexpression of adar1 alleviates fibrosis	Adar1 participates in regulating miRNA-21, PELI1, SPRY2, COL3A1, and SMAD2 in cells	Díaz-Piña et al. (2018)
Idiopathic colitis	Abnormal loss of ADAR1 in T cells can directly lead to spontaneous colitis		N et al. (2018)
Celiac Disease	The expression deficiency of ADAR1 exacerbates the disease		Di Fusco et al. (2023)
Ulcerative Colitis Gut Mucosa	Loss of ADAR1 Induces Panoptosis and Immune Response		Iannucci et al. (2025)
Sepsis	ADAR1 high expression, knocking down significantly enhances inflammation and intestinal damage, reducing survival rate		Liu et al. (2018)
Oxygen deficiency	ADAR1 can improve hypoxia	ADAR1 participates in the editing of F11R and promotes HIF-1α expression	Ma et al. (2019)
Atherosclerosis	ADAR1 aggravates atherosclerosis	Upregulation of ADAR1 and editing of protease S mRNA in cysteine protease	Stellos et al. (2016)
CAD	ADAR1 affects CAD	ADAR1 upregulates NEAT1 levels	Hartner et al. (2009)
Evans syndrome	ADAR1 mutation group exacerbates disease		Hadjadj et al. (2019)
Age dependent neurodegeneration	ADAR1 inhibits diseases		Keegan et al. (2011)
Depression	Adar1 regulates depression from multiple perspectives	Adar1 exerts regulatory effects through miR-432, circ_0000418, and miR-874-3p	(Simmons et al., 2010; Zhang et al., 2021a; Zhang et al., 2021b; Wang et al., 2023)
ALS、FTD	ADAR1 can alleviate neurotoxicity		Wang et al. (2023)
TBI	Reduced expression of ADAR1 after brain injury	Reduced expression of ADAR1 further upregulates circHtra1, promoting neuronal loss	Zheng et al. (2022)
Ischemic stroke	ADAR1 improves ischemic stroke	ADAR1 promotes cell proliferation and reduces the production of inflammatory cytokines	Cai et al. (2023b)
PD	ADAR1 participates in regulating the neuroinflammatory cascade of Parkinson’s disease	Abnormal or dysregulated ADAR1 response and RNA editing may lead to sustained inflammatory response in astrocytes triggered by alpha synuclein aggregation	D'Sa et al. (2025)

ADAR1, through its two principal isoforms p110 and p150, serves as a critical gatekeeper in maintaining innate immune homeostasis by preventing inappropriate recognition of dsRNA (Sun et al., 2021). The interferon-inducible p150 isoform, localized predominantly in the cytoplasm, is the primary defense against cytosolic dsRNA sensors. It suppresses the activation of melanoma differentiation-associated gene 5 (MDA5) through both editing-dependent and editing-independent mechanisms. By catalyzing A-to-I RNA editing on endogenous dsRNAs, ADAR1 p150 destabilizes the duplex structure, thereby masking ‘self’ RNA from MDA5 recognition and preventing the initiation of a deleterious type I interferon response via the MAVS signaling pathway (de Reuver and Maelfait, 2024). Genetic evidence underscores this vital role, as loss-of-function mutations in ADAR1 in humans lead to Aicardi-Goutières syndrome (AGS), a severe autoinflammatory disorder characterized by chronic interferon signaling. Concurrently, ADAR1 p150 also inhibits the dsRNA-activated protein kinase (PKR). While its editing activity can destabilize PKR-activating dsRNAs, p150 can also sequester dsRNAs through direct binding, competitively inhibiting PKR engagement and averting translational shutdown (Parchure et al., 2025). Notably, *in vivo* studies reveal that MDA5 activation is the primary driver of the severe autoinflammatory phenotypes, as its deletion can rescue the lethality observed in ADAR1-deficient mice, whereas PKR deletion cannot. When ADAR1 or p150 subtypes are absent in mice, it can result in embryonic lethality driven by the overexpression of interferon-stimulated genes (Vuillier et al., 2022; Liang et al., 2023).

In contrast, the constitutively expressed p110 isoform is primarily nuclear but can translocate to the cytoplasm under cellular stress. Although its role in directly antagonizing cytosolic dsRNA sensors like MDA5 and PKR is less prominent than that of p150, p110 contributes to cellular survival under stress conditions. It does so by editing and stabilizing specific mRNA transcripts, particularly those containing inverted Alu repeats in their 3′-UTRs, thereby protecting them from Staufen-mediated decay and suppressing stress-induced apoptosis (Licht and Jantsch, 2017; Dorrity et al., 2023). This function represents a distinct, yet crucial, layer of immunoregulation by maintaining cellular integrity and preventing the release of immunostimulatory molecules. ADAR1p110 plays a role in protecting mRNA encoding anti-apoptotic proteins from degradationand maintaining genomic stability in cancer cells (Elbarbary and Maquat, 2017; Shiromoto et al., 2021).

**FIGURE 2:**
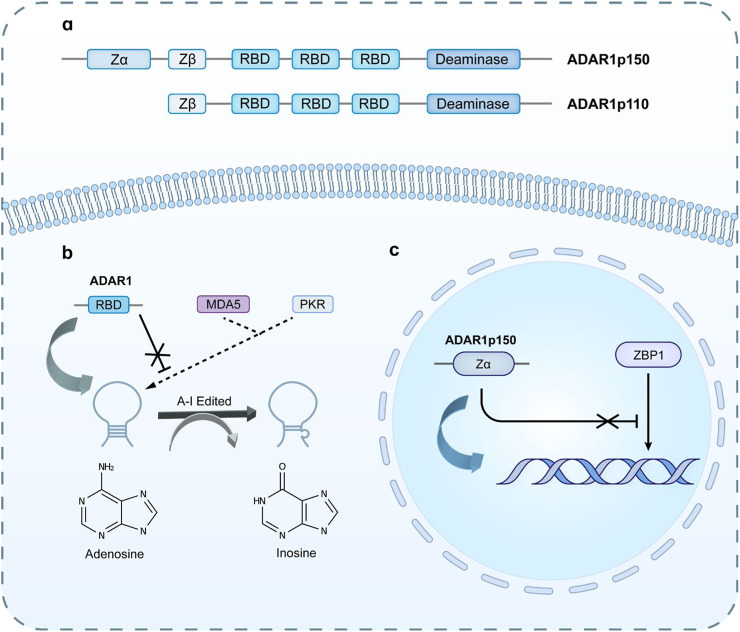
**(a)** Schematic structure of two forms of ADAR1, in which ADAR1p150 containsa Zα domain. **(b)** ADAR1 recognizes dsRNA through RBD and performs A-I editing on it, preventing the recognition of MDA-5 and PKR. **(c)** The unique Zα domain in ADAR1p150 can recognize Z-RNA and edit it, preventing the recognition of ZBP1 and avoiding the occurrence of IFN response.

DsRNA typically adopts a right-handed (A-RNA) conformation and is recognized by the dsRNA binding domain (dsRBD) of ADAR1, thereby preventing the host A-RNA sensors, MDA-5 and PKR, from amplifying immune responses (Mannion et al., 2014; Liddicoat et al., 2015; Pestal et al., 2015; Gannon et al., 2018; Ishizuka et al., 2019; Liu et al., 2019; Mehdipour et al., 2020; Chen et al., 2021a; de Reuver et al., 2021; Maurano et al., 2021; Nakahama et al., 2021; Tang et al., 2021). In addition to binding to A-type dsRNA, the ADAR1p150 subtype also possesses a Zα domain, which can bind to left-handed RNA (Z-RNA) and left-handed DNA (Z-DNA) (Herbert et al., 1997; Herbert, 2020). Z-type nucleic acid binding protein 1 (ZBP1) also possesses a related Zα domain, which enables ZBP1 to bind to Z-RNA and Z-DNA (Zhang T. et al., 2022). ADAR1 can compete with ZBP1 for Z-RNA and Z-DNA, preventing ZBP1 activation and ultimately avoiding serine threonine kinase 3 (RIPK3)-mediated necroptosis (Zhang T. et al., 2022). As shown in Figure 2.

In addition to avoiding recognition by the aforementioned three dsRNA sensors, ADAR1 also functions through the following mechanisms.

### 1.1 OAS/RNase L pathway

**FIGURE 3:**
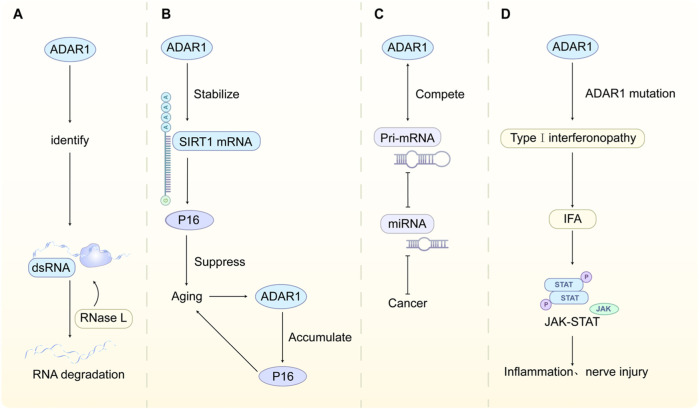
**(A)** ADAR1 edits dsRNA to avoid OAS recognition and prevent cell RNA from being incorrectly identified as pathogen RNA. **(B)** ADAR1 stabilizes SIRT1 mRNA to inhibit p16 expression and delay cellular aging. Aging, in turn, leads to a decrease in ADAR1 protein levels and accumulation of p16. **(C)** ADAR1 competes with DROSHA/DGCR8 complex to bind pri miRNA and regulate the expression of mature miRNA **(D)** The abnormally elevated IFN caused by ADAR1 mutations triggers inflammation and nerve damage through the JAK-STAT pathway.

OAS (oligoadenylate synthetase) recognizes dsRNA and activates RNase L, leading to RNA degradation. ADAR1 inhibits this pathway by editing dsRNA, preventing cellular RNA from being mistakenly identified as pathogen RNA (Daou et al., 2020). As shown in Figure 3A.

### 1.2 Wnt/β-catenin pathway

In acute myeloid leukemia (AML), ADAR1 promotes tumor cell proliferation by regulating key molecules in the Wnt pathway (such as β-catenin, c-Myc, and Cyclin D2). Knockdown of ADAR1 suppresses Wnt signaling and induces cell cycle arrest (Shi et al., 2024).

### 1.3 P16INK4a senescence pathway

ADAR1 inhibits p16 expression by stabilizing SIRT1 mRNA, thereby delaying cellular senescence. During senescence, ADAR1 protein levels decrease, leading to the accumulation of p16 (Hao et al., 2022). As shown in Figure 3B.

### 1.4 MiRNA biogenesis

ADAR1 competes with the DROSHA/DGCR8 complex for binding to pri-miRNA, regulating the expression of mature miRNAs and influencing tumor progression (Cai et al., 2023a). As shown in Figure 3C.

### 1.5 JAK - STAT signaling pathway

In type I interferonopathies caused by ADAR1 mutations, abnormally elevated IFN triggers inflammation and neurological damage through the JAK-STAT pathway. JAK inhibitors (such as Ruxolitinib) can alleviate symptoms (Cattalini et al., 2021). As shown in Figure 3D.

ADAR1 is an essential RNA-editing enzyme that plays a dual role in maintaining physiological health through two distinct molecular mechanisms: a canonical “editing-dependent” pathway that regulates immune responses (Rehwinkel and Mehdipour, 2024), and an “editing-independent” pathway involved in cellular development, differentiation, and organ homeostasis. As previously noted, the editing-dependent immunoregulatory function of ADAR1 is mediated by A-to-I RNA editing on dsRNA, a process widely regarded as critical for enabling cells to distinguish self from non-self RNA. In contrast, through its editing-independent actions, ADAR1 exhibits functional versatility. It can compete with other dsRNA-binding proteins—such as PKR and Dicer—for binding to shared RNA substrates, thereby inhibiting their activities (Ota et al., 2013; Manjunath et al., 2025). Additionally, ADAR1 can serve as a scaffold to interact with various protein partners, or indirectly modulate cellular signaling pathways and gene expression networks through its extensive dsRNA-binding capacity.

Via these editing-independent mechanisms, ADAR1 plays a broad role in the development and differentiation of diverse cell types, including hematopoietic stem cells and neural cells, and is indispensable for the homeostasis of organs such as the liver and spleen. Functioning as a versatile molecular orchestrator, ADAR1 employs the synergy of these two mechanisms to collectively ensure normal development and the maintenance of health. The absence of ADAR1 can affect the normal function of cells and even lead to cell death, depending on the severity of the deficiency. However, when it comes to influencing disease progression, ADAR1 does not exhibit a consistent pattern of exacerbating or alleviating the disease, and it plays varying roles in different diseases. As shown in Figure 1.

## 2 Immune system and immune system diseases

The expression of Adar1 holds significant importance for the development, differentiation, and functionality of immune cells. Adar1 can promote early B cell survival and regulate the transition from early pro-B cells to large pro-B cells via MDA5-dependent mechanisms. Regulating the expression of pre-B cell receptors through MDA5-independent mechanism to control the transition from large pre-B cells to small pre-B cells (Chen et al., 2022). If Adar1 is removed from activated B cells, it will lead to defects in T cell dependent antibody response and a decrease in germinal center B cells (Li et al., 2022). In addition, ADAR1 deficiency results in the preferential systemic loss of CD8/CD103 dendritic cells (Baal et al., 2019) and abnormal maturation of thymic T cells (N et al., 2018),thereby highlighting the crucial role of Adar1 in immune cells.

As early as 2002, it was discovered that ADAR1 plays a role in the development of immune system diseases (Laxminarayana et al., 2002). Initially, high expression levels of ADAR1 were observed in patients with systemic lupus erythematosus (SLE). Notably, the presence of high circulating concentrations of interferon one was found to significantly elevate the levels of ADAR1 mRNA in T cells (Rönnblom and Alm, 2001). Similarly, Adar1 is overexpressed in the synovium of rheumatoid arthritis (RA) patients, and its expression level tends to decrease when RA treatment is effective (Vlachogiannis et al., 2020).

Overexpression of ADAR1 is manifested as exacerbating immune responses. In the study of bone marrow regeneration, a common host immune response following infection, it was discovered that Adar1 promotes infection-induced bone marrow regeneration by binding to and disrupting the stability of Socs3 mRNA in an RNA editing-independent manner (Shen et al., 2018). In addition, genetic analysis indicates that immune dysregulation triggered by Adar1 is one of the significant contributing factors to sudden infant death syndrome (Hafke et al., 2019).

The most common immune system disorder caused by Adar1 mutations is AGS. AGS is a monogenic inflammatory disease associated with elevated levels of type I interferon. Patients with AGS may exhibit neuroinflammatory symptoms, increased interferon-stimulated gene (ISG) signatures in the blood and cerebrospinal fluid (de Reuver and Maelfait, 2024),and suffer from encephalopathy accompanied by white matter malnutrition and intracranial calcification (Inoue et al., 2021). With the confirmation that mutations in Adar1 can lead to the autoimmune disease AGS in mice (Rice et al., 2012), it has been realized that Adar1 plays a significant role in immune system diseases. Multiple case reports consistently suggest that mutations in Adar1 are a susceptibility factor for AGS (La Piana et al., 2014; Straussberg et al., 2015; Kono et al., 2016; Garau et al., 2019; Sathishkumar et al., 2021; Liu et al., 2022; Linares-Navarro et al., 2023; Ahmed et al., 2024; Liu et al., 2024). K999N (Guo et al., 2021), D1113H (Guo et al., 2022a) and p. Gly1007Arg (Rice et al., 2017) are currently confirmed Adar1 mutation sites, capable of inducing the occurrence of AGS.

The most common adverse reaction following organ and cell transplantation is immune rejection (Ochando et al., 2019; Duneton et al., 2022; Li and Lan, 2023), and Adar1 also plays a significant role in regulating this immune response. ADAR1 can directly interact with the miR-21 precursor to facilitate the M2 polarization of mouse splenic macrophages, a process that hyperpolarizes immune cells and prevents their excessive activity, ultimately suppressing allograft rejection (Li et al., 2018). Additionally, by increasing the expression of anti-inflammatory cytokines and promoting M2 polarization of liver macrophages, liver ischemia-reperfusion injury can be alleviated, which is a major risk factor for poor prognosis in patients undergoing liver transplantation (Wang et al., 2024). In mice with acute graft-versus-host disease (aGVHD), overexpression of Adar1, by inhibiting miR-21b, reduced the proportion of Th17 (pro-inflammatory) cells and increased the proportion of Treg (anti-inflammatory) cells. This significantly ameliorated tissue necrosis and reticular structure in the liver and lungs affected by aGVHD, thereby inhibiting the progression of aGVHD following allogeneic hematopoietic stem cell transplantation (Zhao et al., 2023).

Intriguingly, a study exploring chemically induced asthma triggered by low molecular weight compounds did not exclusively concentrate on the immune system. Notably, Diphenylmethane diisocyanate was observed to elevate the expression of type I interferon-stimulated genes in non-sensitized mice during asthma induction, accompanied by an increase in ADAR1 levels (Wisnewski and Liu, 2024). This finding underscores the pivotal role of ADAR1 within the immune system.

Adar1 plays an important role in mice, fruit flies, and also in human immune system diseases. Based on current research, the existence of Adar1 mitigates immune rejection reactions, which relies heavily on the Adar1 editing mechanism. Specifically, Adar1 performs A-to-I RNA editing on its own ligands to prevent the chronic overactivation of dsRNA sensors, namely, MDA5 and PKR (Mannion et al., 2014; Liddicoat et al., 2015; Pestal et al., 2015; de Reuver et al., 2021; Maurano et al., 2021; Nakahama et al., 2021; Tang et al., 2021). However, in other immune system diseases, Adar1 is highly expressed, exacerbating the disease in a non editing dependent manner. In the immune system, Adar1 acts as a double-edged sword, capable of maintaining the homeostasis, development, and differentiation of immune cells, while also exacerbating immune system diseases. Clearly, Adar1 is a valuable target. In-depth research on the mechanism and pathway of Adar1 *in vivo*, and its translation into usable drugs, will make significant contributions to regulating immune cell development, treating immune system diseases, and improving immune rejection reactions in humans.

## 3 Virus

ADAR1’s impact on the immune system directly influences the human body’s ability to resist external viruses. However, a growing body of research has demonstrated that, beyond its effects on the immune system, ADAR1 can also directly influence viral synthesis and replication, as well as the innate immune response triggered by viruses. This article will categorize viral replication strategies and elucidate how ADAR1 directly affects various viruses and their induced innate immune responses.

### 3.1 Class I - double stranded DNA (dsDNA)

ADAR1 is clearly capable of influencing viruses with this most common structural type in nature, including Cowpox virus (VACV), Polyoma virus (PyV), Human Cytomegalovirus (HCMV), Orf virus (ORFV), Kaposi’s sarcoma-associated herpesvirus (KSHV), and Herpes simplex virus 1 (HSV-1), a total of six viruses.

#### 3.1.1 Cowpox virus (VACV)

Vaccinia virus is the prototype and most extensively studied member of the Poxviridae family, a cytoplasmic replication virus family containing a large ∼200 kbp linear dsDNA genome (El-Jesr et al., 2020). In baby hamster kidney BHK-21 cells infected with VACV, the E3L gene of cowpox virus encodes E3 protein, which can activate ADAR1, thereby aiding the virus in evading the host’s immune response (Domingo-Gil et al., 2008). Furthermore, the VACV protein E3 is capable of competing with Z-type RNA on1 through its N-terminal Zα domain, suppressing the accumulation of Z-type RNA that would otherwise occur early stages of VACV infection. This suppression prevents the triggering of ZBP1 recruitment receptor interacting protein kinase three and the execution of necrotic apoptosis, thereby mitigating the host’s innate defense mechanisms against the virus (Koehler et al., 2021). Owing to its capacity to infect diverse cancer cells, the VACV has emerged as a promising candidate for oncolytic virus therapy (Xu et al., 2023).

#### 3.1.2 Polyoma virus (PyV)

When the multi-tumor virus infects mouse embryonic fibroblasts with genetically inactive ADAR1, the virus-induced cell killing is significantly potentiated (George and Samuel, 2011).

#### 3.1.3 Human cytomegalovirus (HCMV)

Upon infection of NK cells by HCMV, the expression of ADAR1-p110 is induced, and subsequently, P110 further elevates the expression of miR-376a. MiR-376a is capable of downregulating the immune regulatory molecule HLA-E, thereby facilitating the elimination of HCMV-infected cells by NK cells (Nachmani et al., 2014).

#### 3.1.4 Orf virus (ORFV)

During the infection of HEK 293T cells by ORFV, OV20.0 directly interacts with the dsRNA binding domain of ADAR1, suppressing its A-to-I RNA editing capability. Conversely, ADAR1 facilitates the expression of OV20.0 and exerts a proviral effect by inhibiting the phosphorylation of PKR (Liao et al., 2019).

#### 3.1.5 Kaposi’s sarcoma associated herpesvirus (KSHV)

KSHV exhibits two distinct life cycles during infection: latency and lytic reactivation. Research has indicated that ADAR1 functions as a proviral factor for KSHV lysis and reactivation. Knocking down ADAR1 in KSHV-latently infected cells suppresses viral gene transcription and replication during KSHV lysis and reactivation, and concurrently, significantly enhances the production of type I interferons during KSHV reactivation (Zhang et al., 2020).

#### 3.1.6 Herpes simplex virus 1 (HSV-1)

ADAR1 p150 interacts with PKR to inhibit PKR/eIF2α - mediated translation arrest triggered by HSV-1 infection and promote viral replication (Parchure et al., 2025).

### 3.2 Class IV virus - sense single stranded RNA virus (ssRNA)

Positive-sense single-stranded RNA (+ssRNA) viruses are the most prevalent eukaryotic viruses in nature, requiring their genomic (positive-sense) RNA (+RNA) to serve as a replication template for synthesizing negative-sense RNA (-RNA) (Gong et al., 2023). ADAR1 can influence the replication and spread of Hepatitis C virus (HCV), Dengue virus (ENV), Severe Acute Respiratory Syndrome Coronavirus 2 (SARS-CoV-2), Coxsackievirus (CVB3), Zika virus (ZIKV), and Human enterovirus D68 (EV-D68), some of which pose a significant threat to global human health. ADAR1 may serve as a breakthrough in.

#### 3.2.1 Hepatitis C virus (HCV)

Hepatitis C virus infection poses a significant global health concern, affecting approximately 57 million individuals worldwide with chronic viremia, leading to 290,000 deaths and 1.5 million new infections annually (Polaris Observatory H CV Collaborators, 2022; Yang et al., 2021). As the veil of mystery surrounding ADAR1 is gradually lifted, it is anticipated to emerge as a promising breakthrough in the treatment of HCV. In 2005, Taylor DR et al. initially reported that ADAR1 participates in a potent antiviral pathway, specifically eliminating HCV RNA through A-to-I RNA editing (Taylor et al., 2005). Over the years, the hepatitis C virus has developed mechanisms to suppress and evade innate immunity, leading to significant variations in the response to IFN therapy among patients with chronic HCV infection (Pujantell et al., 2018). Notably, when administering pegylated interferon (PEG-IFN) and ribavirin to African American patients infected with HCV, eradicating the infection remains an elusive goal (Luo et al., 2005).

In a study examining the association between polymorphism in the ADAR1 gene and the severity of liver disease in European patients co-infected with HIV/HCV, it was discovered that five SNPs within the ADAR1 gene (rs1127326, rs1127317, rs1127314, rs1127313, and rs2229857) can decrease the rate of fibrosis progression, shield carriers from advanced fibrosis, enhance the aspartate aminotransferase to platelet ratio index and FIB-4 index, and avert the onset of severe liver disease (Med et al., 2017). HCV continues to exhibit inherent resistance to current treatment regimens (Vo-Quang and Pawlotsky, 2024). As research on Adar1 deepens, it may become possible to devise novel treatment strategies to overcome this resistance.

#### 3.2.2 Dengue virus (DENV)

Dengue virus, a positive-stranded RNA virus belonging to the Flaviviridae family, serves as the causative agent of dengue fever. Dengue fever is a viral illness primarily transmitted by mosquitoes. Every year, millions of people are infected with the virus through the bites of infected female Aedes mosquitoes (Sinha et al., 2024). ADAR1 serves as a pro-viral host factor that facilitates the replication of dengue virus. Upon infection, the NS3 protein of the virus interacts with ADAR1, thereby enhancing its editing activity and subsequently promoting the synthesis and replication of viral proteins (de Chassey et al., 2013). Furthermore, during the early stages of DENV infection, ADAR1 is modulated by miR-3614-5p, thereby enhancing the infectivity of DENV (Diosa-Toro et al., 2017).

While the current quadrivalent dengue fever vaccine has demonstrated long-term efficacy and safety, its effectiveness ranges only from 60% to 90% (Tricou et al., 2024; Kallás et al., 2024), falling short of providing highly effective protection against DENV across all age groups. ADAR1 has been confirmed to enhance the infectivity of DENV. Developing a novel vaccine that can suppress the expression of this enzyme on the current foundation, or creating new therapeutic agents centered on ADAR1, may potentially boost treatment effectiveness and tackle this global dilemma.

#### 3.2.3 Severe Acute Respiratory Syndrome Coronavirus 2 (SARS-CoV-2)

Severe Acute Respiratory Syndrome Coronavirus 2 is a member of the Severe Acute Respiratory Syndrome related coronavirus species and the sole representative of the Sarbecovirus subgenus. Predominantly found in horseshoe bats (Coronaviridae Study Group of the International Committee on Taxonomy of Viruses, 2020), it marks the third highly pathogenic coronavirus to emerge in humans within the past 2 decades (Zhu et al., 2020; Zhou et al., 2020).

ADAR1 is capable of mediating the A-to-G editing within the SARS-CoV-2 genome (Picardi et al., 2021), and its activity is potentiated upon infection with the virus (Light et al., 2021). Suppressing ADAR1 results in a diminished editing level in human alveolar cells (Peng et al., 2022). The ablation of Adar1 in myeloid cells can establish a persistent antiviral state in the lungs, enhancing early immunity to SARS-CoV-2 (Adams et al., 2023). During the replication of SARS-CoV-2, ADAR1 can cause mutations in certain sections of the genome, exerting antiviral effects (Gregori et al., 2021). However, during the editing process of SARS-CoV-2, ADAR1 similarly expedited its evolution in humans (Song et al., 2022).

The swift dissemination and ongoing evolution of novel variants of SARS-CoV-2 pose a persistent threat to global public health (Steiner et al., 2024). By October 2025, SARS-CoV-2 had resulted in over 777 million confirmed infections and claimed the lives of more than seven million individuals (World Health Organization). While ADAR1 can indeed expedite evolution, its presence also serves as a defense against SARS-CoV-2, potentially paving a new path for the enhancement of vaccines and the development of therapeutic agents.

#### 3.2.4 Coxsackievirus (CVB3)

ADAR1 demonstrates a dual-edged effect in CVB3-induced viral myocarditis. During the early stage of infection, the downregulation of ADAR1 suppresses viral replication and inflammatory response, thereby improving CVB3-induced viral myocarditis (VMC). However, in the middle and late stages, the downregulation of ADAR1 exacerbates disease progression by promoting inflammatory response via PKR and NF-κB signaling (Dong et al., 2016).

#### 3.2.5 Zika virus (ZIKV)

Zhou S et al. discovered in their research that ADAR1 exerts proviral effects by suppressing IFN production and PKR activation. Both ADAR1 knockout and knockdown significantly decreased ZIKV RNA synthesis, protein levels, and viral titers in human lung cancer epithelial cells, human embryonic kidney cells, and glioblastoma cells (Zhou et al., 2019).

#### 3.2.6 Human enterovirus D68 (EV-D68)

Zhang K et al. discovered that the transcription and expression of ADAR1 are suppressed following EV-D68 infection. Overexpression of ADAR1p110 facilitates viral replication by directly editing EV-D68 RNA and activating its deaminase domain’s active site (Zhang K. et al., 2022).

### 3.3 Class V virus - negative single stranded RNA virus (NSRVs)

Negative-sense single-stranded RNA viruses possess a ribonucleoprotein (RNP) complex composed of viral polymerase and genomic RNA encased by viral nucleoprotein (Zhou et al., 2013). A review of all recent studies reveals that ADAR1 can exert effects on six negative-sense single-stranded RNA viruses: Influenza A virus (IAV), Vesicular stomatitis virus (VSV), Lymphocytic choriomeningitis virus (LCMV), Measles virus (MeV), Ebola virus (EBOV), and Marburg virus (MARV).

#### 3.3.1 Influenza A virus (IAV)

The influenza A virus (IAV) is an enveloped, negative-stranded, single-stranded RNA virus that can cause mild to severe respiratory diseases (Haas et al., 2023). During IAV infection, Adar1 facilitates virus synthesis and replication by targeting NS1, a multifunctional protein and virulence factor whose expression is crucial for optimal virus protein synthesis and replication (de Chassey et al., 2013). Paradoxically, ADAR1 can effectively restrict influenza viruses (Suspène et al., 2011). There are multiple subtypes of IAV. When human epithelial cells are infected by H1N1 and H3N2 subtypes of IAV, they exhibit upregulation of ADAR1 and increased A-to-I RNA editing activity (Cao et al., 2018). During IAV infection, ADAR1 enhances the activation of Toll-like receptors 7 and 8 (TLR7/8) through A-to-I RNA editing of the ssRNA of influenza A virus, triggering an innate immune response (Sarvestani et al., 2014). With the research conducted by Vogel OA and his colleagues, the nature of ADAR1 has been revealed. The two subtypes of ADAR1 play opposite roles during IAV infection, with the p110 subtype restricting and the p150 subtype promoting IAV replication (Vogel et al., 2020). Disrupting the p150 subtype can prevent the cytopathic effects caused by IAV (Ward et al., 2011).

IVA causes significant economic losses every year (Putri et al., 2018),spreading in the form of seasonal infections among humans and animals, and in severe cases leading to hospitalization and death (Kelly et al., 2011). ADAR1 can both promote the synthesis and replication of IAV proteins and activate innate immune responses to restrict IAV. Delving deeper into the relationship between ADAR1 and IAV, and exploring unknown targets, pathways, and interactions, could represent a novel research direction towards completely overcoming IAV.

#### 3.3.2 Vesicular stomatitis virus (VSV)

Vesicular stomatitis virus belongs to the Vesicular Viridae family and the Vesicular Virus genus. It is a well-studied livestock pathogen and a prototype non-segmented, negative-stranded RNA virus (Liu et al., 2021). In a study examining the relationship between Adar1 and VSV, researchers uncovered an intriguing finding: Adar1 enhances VSV replication via a mechanism that is independent of dsRNA editing. Specifically, it only necessitates the N-terminal domain, without the need for the deaminase domain, to inhibit PKR activation (Nie et al., 2007; Li et al., 2010). Moreover, in HeLa cells infected with VSV, the stable knockdown of ADAR1 enhances their responsiveness to IFN therapy (Li et al., 2010). Currently, VSV is commonly used as an oncolytic virus for anti-tumor therapy (Evgin et al., 2022; Kottke et al., 2021). Theoretically, by combining it with the inhibition of ADAR1 expression, it can more effectively treat tumors. Developing a therapeutic approach that can be combined with VSV to stably inhibit ADAR1 expression can greatly assist in the treatment of tumors.

#### 3.3.3 Lymphocytic choriomeningitis virus (LCMV)

Lymphocytic choriomeningitis virus is a non-cytopathic virus belonging to the genus of arenaviruses (Stachura et al., 2023). Mice infected with LCMV show a significant upregulation in the expression and activity of ADAR1-L. ADAR1-L has the ability to induce mutations in viral RNA, resulting in the loss of viral protein function and decreased viral infectivity (Zahn et al., 2007). ADAR1 is upregulated following LCMV infection to diminish viral infectivity, further proving its significant role in the immune response. LCMV, similar to the VSV mentioned earlier, is also utilized in anti-tumor therapy owing to its capacity to elicit potent immune responses (Purde et al., 2024; Mariniello et al., 2024; Gomar et al., 2024).

#### 3.3.4 Measles virus (MeV)

Measles virus belonging to the family Paramyxoviridae, is a highly contagious enveloped virus with a single-stranded negative RNA. It is primarily transmitted through the respiratory tract (Katz and Hinman, 2004; Fiebelkorn et al., 2017). MeV demonstrates a high degree of sensitivity to the constraints imposed by ADAR1 (Suspène et al., 2011). Although ADAR1p150 infrequently edits the tightly packaged genomes of standard measles virus, it is capable of effectively editing genomes with inverted repeat sequences that are unintentionally produced by mutant MeV (Pfaller et al., 2018). Editing has the potential to modify the MEV structure, ultimately leading to the suppression of innate immune responses (Pfaller et al., 2021).

After HeLa cells are infected with the wild-type measles virus, ADAR1 can effectively suppress the viral infection by enhancing cell apoptosis (Toth et al., 2009), activating PKR and IRF3, inducing IFN-β (Li et al., 2012) and suppressing the stress granule response (Okonski and Samuel, 2013). ADAR1 serves as a limiting factor for MeV in mouse embryonic fibroblasts (MEF) as well. ADAR1 P150-deficient MEFs exhibit extensive syncytial formation and cytopathic effects upon MeV infection, whereas the restoration of ADAR1 effectively prevents MeV-induced cellular pathology (Ward et al., 2011).

The measles virus poses a significant threat to public health, directly resulting in over 100,000 deaths each year (Mina et al., 2019). Despite the availability of safe and effective MeV vaccines, they still result in a significant number of cases and fatalities (Fiebelkorn et al., 2017), and currently, there are no approved drugs for MeV treatment (Zyla et al., 2024). Given the suppressive effect of ADAR1 on MeV, it will be exploited as a novel target for the development of drugs aimed at achieving a complete cure for MeV.

#### 3.3.5 Ebola virus (EBOV) and marburg virus (MARV)

ADAR1 can edit the 3 UTRs (negative regulators of translation) in the genome of EBOV and MARV, reducing their impact. In addition, the innate immune response induced by ADAR1 can inactivate the mRNA 3 UTR (Khadka et al., 2021).

### 3.4 Class VI - (+) sense single stranded RNA retrovirus

Sense single-stranded RNA retroviruses refer to a class of single-stranded RNA viruses that inherently carry RNA reverse transcriptase, enabling them to reverse transcribe RNA strands into DNA. Studies have confirmed that ADAR1 can significantly influence the replication and infection processes of Human Immunodeficiency Virus Type 1 (HIV-1) and Human T-cell lymphotropic virus (HTLV).

#### 3.4.1 Human immunodeficiency virus type 1

Human immunodeficiency virus type 1, first discovered in 1983 (Barré-Sinoussi et al., 1983), is a lentivirus thatintegrates into the genome of host cells (Ahmed and Herschhorn, 2024), ultimately leading to the development of acquired immunodeficiency syndrome (AIDS) (Gallo et al., 1984; Popovic et al., 1984). Numerous studies have shown that ADAR1 can stimulate human immunodeficiency virus type 1 replication in an editing dependent manner (binding to HIV-1 RNA and editing adenosine and Rev and Tat coding sequences in the five untranslated regions) (Doria et al., 2009), and knocking down Adar1 can inhibit HIV-1 replication and expression in various cells *in vivo*.

As previously mentioned, individuals with ADAR1 deficiency are predisposed to developing the congenital immune disorder AGS. Intriguingly, this same deficiency has the ability to impede HIV-1 replication in CD4+T lymphocytes of AGS patients at the protein translation level (Cuadrado et al., 2015). In human embryonic kidney cells, overexpression of ADAR1 enhances the expression of HIV-1 structural proteins and viral production, whereas its downregulation suppresses HIV-1 expression (Phuphuakrat et al., 2008). In HEK 293T cells, ADAR1 reverses the suppressive effects of PKR on HIV expression and production by interacting with TAR RNA binding protein (Clerzius et al., 2009). In astrocytes, the expression of adar1 is upregulated following HIV-1 infection, and the administration of small interfering RNAs specifically targeting ADAR1-p150 results in a moderate reduction in HIV-1 production (Clerzius et al., 2009). Similarly, knocking down adar1 in primary macrophages can potentiate the production of interferon, cytokines, and chemokines. These macrophages function as antiviral paracrine factors, rendering them resilient to HIV-1 infection (Pujantell et al., 2017).

Over the past 4 decades since the discovery of HIV-1, the diverse mechanisms of immune evasion have posed significant challenges in the development of effective HIV-1 vaccines (Ahmed and Herschhorn, 2024), and immune mechanisms may become a successful method for eradicating or curing HIV-1 infection (Armani-Tourret et al., 2024). ADAR1 plays a crucial role in innate immune response, and knocking down its expression can exert inhibitory effects on HIV-1. As a target that remains underexplored and untapped, Adar1 continues to hold significant potential for further investigation. Starting from ADAR1 to explore new vaccines and therapies could indeed be a novel direction and approach worth considering.

#### 3.4.2 Human T-cell lymphotropic virus

ADAR1 can potentiate HTLV-1 and HTLV-2 infection in T lymphocytes through a non-editing-dependent mechanism. ADAR1 can further suppress the inhibitory effect of IFN-α on HTLV-1 and HTLV-2 by inhibiting the phosphorylation of PKR (Cachat et al., 2014).

### 3.5 Class VII - double stranded DNA retrovirus

Double stranded DNA retrovirus is a kind of virus whose genome is DNA, but it must undergo an RNA intermediate and reverse transcription step in its replication cycle. This represents a highly distinctive and unique category of viruses. Nevertheless, ADAR1 can exert an impact on hepatitis B virus (HBV) and hepatitis D virus (HDV), the latter of which relies on HBV for infection and transmission.

#### 3.5.1 Hepatitis B virus (HBV)

Hepatitis B virus is a small double-stranded DNA virus (Yu et al., 2024), Chronic infection can lead to cirrhosis and hepatocellular carcinoma, resulting in nearly one million deaths every year (Nash and Davies, 2024). ADAR1 can inhibit the expression of MAVS through post-transcriptional regulation mediated by human antigen R (HuR). MAVS exerts antiviral activity both *in vitro* and *in vivo*, and reduces the levels of HBV markers, thereby exerting an antiviral effect (Li et al., 2021). It is not difficult to explain why the ADAR1 mRNA levels in patients with chronic hepatitis B (CHB) are lower than those in healthy individuals (Wu et al., 2014).

Non-alcoholic fatty liver disease (NAFLD) is a global metabolic disorder (Lin X. et al., 2024) affecting 38% of the world’s population (Wong et al., 2023). Compared to CHB patients, the expression of ADAR1 in the liver tissue of CHB patients with NAFLD is further downregulated (Yang et al., 2022).

HBV infection poses a global public health challenge, ranking alongside tuberculosis, HIV, and malaria in terms of scale (Revill et al., 2019). Although there are effective methods to inhibit virus replication, there is currently no complete cure (Yu et al., 2024). Targeting ADAR1 as a potential biomarker and therapeutic target for HBV, aiming to enhance the clearance of HBV by immune cells and improve the outcome of chronic infection.

#### 3.5.2 Hepatitis D virus (HDV)

The hepatitis D virus is classified as the Deltavirus genus in the Kolmioviridae family, and eight genotypes have been identified (Liu et al., 2023). Its genome is a circular single-stranded RNA, consisting of approximately 1700 nucleotides. It is the known pathogenic virus that has the minimal impact on humans (Sureau and Negro, 2016) and requires co-infection with HBV to enter liver cells (Negro and Lok, 2023). JJayan GC et al. found in their study that overexpression of ADAR1 inhibits HDV RNA replication and impairs viral activity (Jayan and Casey, 2002). Wong SK et al. further confirmed in their study that the small form of ADAR1 (ADAR1-S), which resides in the nucleus, edits HDV RNA during replication, and interfering with ADAR1-S can effectively inhibit HDV replication (Wong and Lazinski, 2002). Certainly, the larger form of ADAR1 (ADAR1-L), which is localized in the cytoplasm, possesses the capability to edit HDV RNA. However, the HDV genome incorporates an inherent negative feedback regulatory mechanism that restricts editing to approximately one-third of its sequence (Hartwig et al., 2004).

**FIGURE 4:**
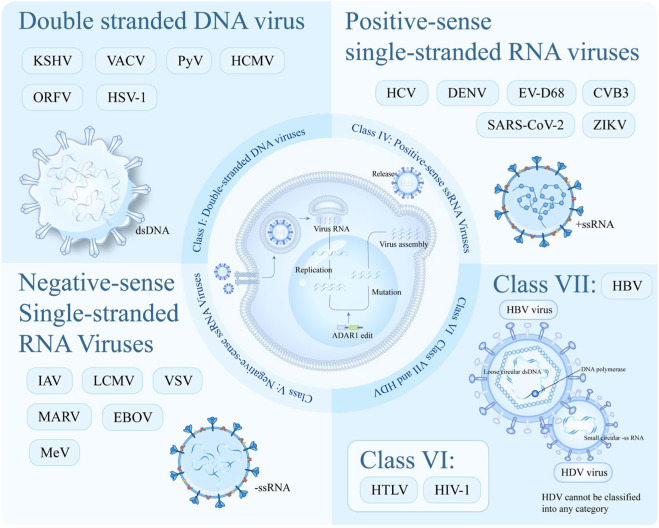
ADAR1 affects a variety of viruses.

HDV is a satellite RNA virus that requires HBV for assembly and reproduction. Individuals infected with HDV progress to advanced liver disease more rapidly than those infected with HBV alone (Polaris Observatory Collaborators, 2024). Adar1 has the ability to activate the innate immune response, thereby inhibiting the proliferation of HDV and HBV. This makes it an excellent target for suppressing the virus and improving end-stage outcomes. As shown in Figure 4.

## 4 Heart and heart disease

Adar1 plays a role in the human life process, not only in the immune system and antiviral response, but also in the normal life activities and disease progression across multiple systems in the human body. Adar1 plays a crucial role in maintaining the survival of myocardial cells and cardiac function (Zhang et al., 2019). It enriches pathways related to myocardial cell differentiation, myocardial contraction, and heart diseases by specifically editing the heart (Chen J. et al., 2021). ADAR1 is capable of preventing the IRF7-mediated cardiac autoinflammatory response triggered by endogenous non RNA in mouse cardiomyocytes. Its absence can lead to the progression of delayed autoinflammatory cardiomyopathy into dilated cardiomyopathy and heart failure at 6 months (Garcia-Gonzalez et al., 2022). In adult mice, the absence of Adar1 results in severe ventricular remodeling and rapid spontaneous cardiac dysfunction (Zhang et al., 2019).

**FIGURE 5:**
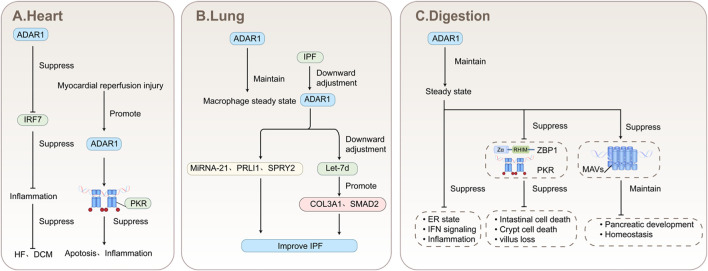
Schematic diagram of the mechanism by which adar1 acts on the heart, lungs, and digestive system **(A)** ADAR1 downregulates the expression of IRF7, further inhibits inflammation, and inhibits the progression of heart failure and dilated cardiomyopathy. Myocardial reperfusion injury upregulates ADAR1, inhibits the expression of PKR, and further inhibits apoptosis and inflammation. **(B)** ADAR1 is involved in regulating pulmonary macrophage homeostasis. The expression of ADAR1 is downregulated in idiopathic pulmonary fibrosis, and the overexpression of ADAR1 can improve symptoms by regulating miRNA-21, peli1, spry2 and let-7d. **(C)** ADAR1 is directly involved in maintaining homeostasis and inhibiting disease by preventing the recognition of zbp1, PKR, and MAVs.

ADAR1 also affects the development of various heart diseases. The expression of ADAR1p150 was markedly upregulated in mouse cardiomyocytes afflicted with viral myocarditis (Zhang et al., 2019). By simulating reactive oxygen species generated during myocardial reperfusion injury using H_2_O_2_, it was discovered that H_2_O_2_ can trigger the production of ADAR1 in neonatal cardiomyocytes, thereby inhibiting the activation of PKR and mitigating its capacity to promote cell apoptosis and inflammation (Wang Y. et al., 2016). As shown in Figure 5A.

Heart disease encompasses a wide range of cardiac conditions, including myocardial reperfusion injury and viral myocarditis mentioned previously, and it remains one of the most prevalent causes of death globally. Currently, all heart diseases involving Adar1 share a common trait: the elevation of Adar1 levels triggered by the disease itself, which subsequently aggravates the condition. However, the presence of Adar1 plays a crucial role the homeostasis of myocardial cells and ensuring the normal structural and functional integrity of the heart. Balancing the beneficial effects of ADAR1 expression on normal cells and organs with the suppressive impact of ADAR1 knockdown on diseases is crucial for rational exploitation of this target. Currently, the research pertaining to the association between ADAR1 and the heart remains inadequate, necessitating further exploration into its pivotal role.

## 5 Liver and drug metabolism

The liver serves as a crucial organ in the human body, overseeing the regulation of the immune system (Kawashima et al., 2024) and metabolism (Lin J. et al., 2024). ADAR1 is crucial for the fetal liver stage of mouse embryos (Hartner et al., 2009), and its absence results in death between the 11.5th and 12.5th day of embryogenesis, accompanied by a rapid breakdown of the liver structure (Hartner et al., 2004). The genetic polymorphism of ADAR1 can effectively prevent severe liver disease in patients with HIV/HCV coinfection (Med et al., 2017), while rs4845384 within ADAR1 is also linked to the treatment-induced clearance of chronic hepatitis B (Wu et al., 2012).

ADAR1 is an important molecule that maintains the homeostasis of the adult liver. When ADAR1 is specifically knocked out of the liver of adult mice, severe structural and functional damage occurs in the liver of the mice (Wang et al., 2015). Furthermore, ADAR1 plays a pivotal regulatory role in maintaining liver immune homeostasis by suppressing crosstalk between NF-κB and type I interferon signaling cascades, thereby mitigating the progression of liver inflammation and fibrosis (Ben-Shoshan et al., 2017).

The liver plays a pivotal role in maintaining metabolic homeostasis (Lin J. et al., 2024), and ADAR1 exerts its influence on hepatic drug metabolism by modulating cytochrome P450 enzymes and the pregnane X receptor. Cytochrome P450 enzymes, also known as P450 or CYP, were first discovered in the early 1960s (Omura and Sato, 1962) and hold significant importance in drug metabolism (Conney, 1967). Approximately 80% of small-molecule drug metabolism is catalyzed by P450 enzymes (Guengerich, 2024). In the liver, ADAR1 functions as a regulator of the P450 enzyme system. HepaRG cells with ADAR1 knockdown exhibit reduced expression of CYP2B6 and CYP2C8 mRNA and protein, along with an elevation in CYP3A4 mRNA levels (Nozaki et al., 2019). The pregnane X receptor (PXR) is a key transcription factor that regulates the expression of various drug metabolism enzymes and transporters. Notably, HepaRG cells with ADAR1 knockdown demonstrate increased levels of PXR mRNA and protein, further influencing hepatic drug metabolism (Takemoto et al., 2021).

**FIGURE 6:**
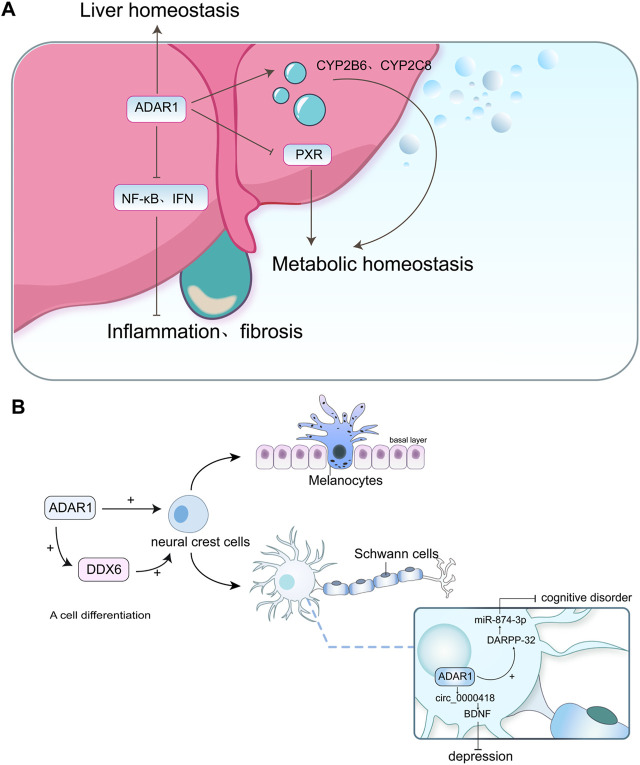
**(A)** ADAR1 directly participates in maintaining liver homeostasis and alleviates the progression of liver inflammation and fibrosis by inhibiting the NF-κB and IFN pathways, while also influencing hepatic drug metabolism by regulating CYP2B6, CYP2C8, and PXR. **(B)** ADAR1 affects the development and differentiation of neural system cells either directly or through DDX6, and its expression can also influence depression and cognitive dysfunction.

ADAR1 also plays an important role in liver diseases. A study on liver ischemia/reperfusion injury (IRI) revealed that suppressing ADAR1 significantly exacerbated inflammation and liver damage following IRI, concomitantly activating the IFN response (Wang H. et al., 2016). The upregulation of ADAR1 impairs the activation of NLRP3 inflammasome and alleviates liver disease in mice with non-alcoholic fatty, which is an inflammatory condition (Xiang et al., 2022). As shown in Figure 6A.

ADAR1 is involved in liver development, maintaining liver homeostasis, regulating liver drug metabolism, and contributing to disease progression, yet there remain numerous uncharted territories deserving further exploration. In the future, ADAR1 could potentially emerge as a novel therapeutic target, facilitating the improved regulation of liver homeostasis and disease management.

## 6 Lung and respiratory system diseases

ADAR1 affects the homeostasis of lung cells, and a deficiency in ADAR1 can directly result in the depletion and dysfunction of alveolar macrophages (Baal et al., 2019). ADAR1 is also implicated in the progression of lung diseases. Interferon induced ADAR1 is involved in the pathogenesis of microvascular lung injury (Rabinovici et al., 2001). Its expression can accelerate acute lung injury by mediating the expression of pro-inflammatory and anti-inflammatory cytokines, as well as influencing tissue neutrophil recruitment and D (A-a)O2 (Wu et al., 2009). In addition, ADAR1 can further aggravate the progression of pulmonary arterial hypertension by mediating the m1A modification of circCDK17 and subsequently triggering the proliferation of pulmonary artery smooth muscle cells (Zhang et al., 2023).

Idiopathic pulmonary fibrosis (IPF) is a rare progressive disease that can cause persistent cough, exertional dyspnea, impaired quality of life, and even death (Lancaster et al., 2024). IPF has a high mortality rate, unpredictable progression, and limited treatment options (Raghu et al., 2024). During the investigation of ADAR1 and IPF, it was observed that ADAR1 was significantly downregulated in IPF fibroblasts. Notably, overexpression of ADAR1 was capable of restoring the expression levels of miRNA-21, PELI1, and SPRY2 in IPF patient fibroblasts. miRNA-2 targets anti-fibrotic molecules, namely, PELI1 and SPRY2. Consequently, overexpressing ADAR1 in IPF patients presents a potential therapeutic strategy (Díaz-Piña et al., 2018). However, it is worth noting that ADAR1 can also upregulate the expression of COL3A1 and SMAD2, proteins implicated in the progression of IPF, by triggering a reduction in the levels of mature Let-7d (microRNA) (Díaz-Piña et al., 2022). Overexpression of ADAR1 can both inhibit and promote the progression of IPF, which is clearly contradictory. As shown in Figure 5B. The precise role of ADAR1 in IPF remains worthy of further investigation. As a refractory disease, targeting ADAR1 may not be a bad choice for IPF.

## 7 Digestive system

The digestive system is one of the eight major systems in the human body, comprising multiple organs such as the stomach, intestines, and pancreas. ADAR1 also plays a role in maintaining homeostasis in the digestive system. The absence of ADAR1 in adult mouse Lgr5+ cells triggers rapid apoptosis and depletion of active cycling stem cells in the small intestine and colon (Qiu et al., 2013). Additionally, it induces endoplasmic reticulum stress and activates IFN signaling, ultimately leading to intestinal inflammation. In addition, ADAR1 can prevent intestinal cell death, crypt cell death, and villus loss by inhibiting the spontaneous activation of the dsRNA sensor ZBP1 and PKR *in vivo*. It can also maintain pancreatic development and homeostasis by inhibiting abnormal activation of the Mavs mediated innate immune pathway (de Reuver et al., 2022; Sinigaglia et al., 2024; Rupani et al., 2023).

Multiple studies have confirmed that the expression of Adar1 affects the progression of digestive system diseases. Abnormal loss of ADAR1 in T cells can directly lead to the development of digestive disease - spontaneous colitis (N et al., 2018). The expression deficiency of ADAR1 can amplify the pathogenic response in the intestinal mucosa of celiac disease, induce Panoptosis and Immune Response in Ulcerative Colitis Gut Mucosa (Di Fusco et al., 2023; Iannucci et al., 2025). ADAR1 has been discovered to be overexpressed in purulent macrophages and small intestinal tissues of mice suffering from sepsis. Knocking down its expression significantly exacerbates inflammation and intestinal damage, leading to decreased survival rates (Liu et al., 2018). However, in mice suffering from gastric metaplastic injury, while the ADAR1-mediated response to dsRNA was significantly elevated, the presence of ADAR1 paradoxically triggered the proliferation and metaplasia of differentiated epithelial cell populations, thereby heightening the risk of carcinogenesis (Sáenz et al., 2022). In the digestive system, ADAR1 can serve as a valuable indicator for evaluating the risk of colitis-associated colorectal tumors in patients diagnosed with ulcerative colitis (Takahashi et al., 2023). As shown in Figure 5C.

## 8 Hematopoietic system and vascular diseases

**FIGURE 7:**
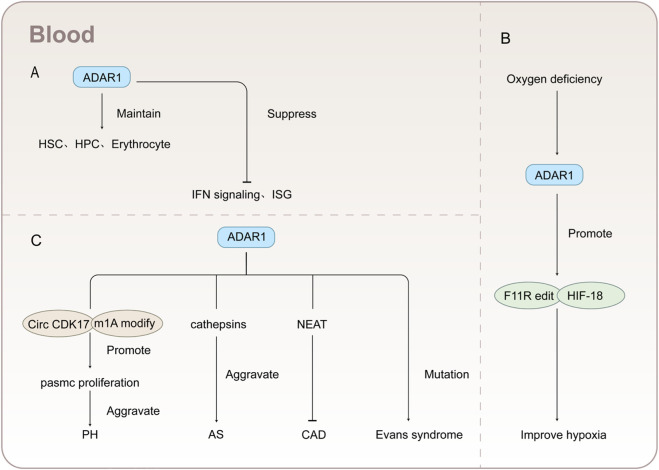
Schematic diagram of the mechanism of adar1 acting on hematopoietic system and vascular diseases **(A)** ADAR1 is directly involved in maintaining cellular homeostasis and inhibiting IFN signaling and ISG. **(B)** In hypoxia, ADAR1 ameliorates hypoxia by enhancing the editing of F11R and upregulating hif-18. **(C)** ADAR1 alters disease progression in multiple ways.

The hematopoietic system is the most crucial system in the human body, fulfilling the daily requirements of various organs for nutrition and oxygen (Kandarakov et al., 2022). ADAR1 is indispensable for maintaining and facilitating the self-renewal of hematopoietic stem cells (HSCs) in fetuses and adults (Hartner et al., 2009; Rossetti et al., 2017). In addition, ADAR1 is also essential for the survival of hematopoietic progenitor cells in adult mice (XuFeng et al., 2009), inhibiting IFN signaling in hematopoietic stem cells and progenitor cells in the body (Hartner et al., 2009). The interaction between ADAR1 and Z-RNA similarly exerts an influence on hematopoietic cells. When a mutation occurs in the ADAR1 Zα nucleic acid binding domain, it loses its capacity to bind to Z-RNA, resulting in an elevation of IFN-stimulated gene expression in hematopoietic cells (Tang et al., 2021). ADAR1 can also exert an impact on the generation of embryonic and normal red blood cells (Wang et al., 2000; Liddicoat et al., 2016), with the ADAR1 ± chimeric embryonic hematopoietic system exhibiting pronounced deficiencies (Wang et al., 2000). Moreover, ADAR1 also contributes to the development of the liver, bone marrow, spleen, and thymus in mice (Hartner et al., 2004). As shown in Figure 7A.

ADAR1 serves as a hypoxia regulatory factor, involved in the editing of F11R, a cell adhesion molecule residing on the surface of human platelets, which plays a pivotal role in platelet aggregation, cell migration, and cell proliferation during hypoxic conditions (Ben-Zvi et al., 2013). ADAR1 can further enhance the induction of target genes and downstream angiogenesis by facilitating the accumulation of HIF-1α following oxygen depletion, thereby alleviating hypoxic conditions (Ma et al., 2019). As shown in Figure 7B.

ADAR1 also participates in regulating vascular endothelial cells. Endothelial cells occupy a pivotal interface between circulating blood and semi-solid tissues, playing crucial safeguarding various physiological functions throughout the body (Bloom et al., 2023). Guo et al. have confirmed that ADAR1 is involved in editing endothelial cell RNA, preventing its recognition by MDA-5 and thus suppressing the occurrence of interferon response (Guo et al., 2022b). When ADAR1 is specifically deleted in mouse endothelial cells, it results in a decreased editing level of short interspersed nuclear element RNA, ultimately leading to neonatal mortality and pronounced damage to multiple organs in mice (Guo et al., 2022b). Furthermore, ADAR1 plays a pivotal role in maintaining the survival, stability, and elasticity of vascular smooth muscle. Mice lacking ADAR1 exhibited apoptosis of vascular smooth muscle cells, mitochondrial dysfunction, extensive bleeding in multiple organs, and deleterious vascular damage, ultimately leading to their demise on day 14 (Cai and Chen, 2024).

ADAR1 is directly involved in the development of vascular diseases. In addition to promoting pulmonary arterial hypertension as mentioned earlier (Zhang et al., 2023),The overexpression of ADAR1 is also manifested in the upregulation of the editing of cathepsin S mRNA, a cysteine protease associated with atherosclerosis, leading to an increase in its protein expression and thereby promoting the progression of atherosclerosis (Stellos et al., 2016). In addition, ADAR1 affects coronary artery disease by upregulating NEAT1 levels (Hartner et al., 2009). During the investigation of Evans syndrome, a rare and severe autoimmune disorder characterized by the combination of autoimmune hemolytic anemia and immune thrombocytopenia, it was observed that individuals with ADAR1 mutations exhibited a more severe manifestation of the disease (Hadjadj et al., 2019). As shown in Figure 7C.

## 9 Neurons and nervous system

Human neurons inherently possess immunostimulatory dsRNA at exceptionally high levels (Dorrity et al., 2023), and the presence of ADAR1 can edit dsRNA to inhibit the occurrence of IFN response. Neuronal progenitor cells with ADAR1 knockout demonstrate spontaneous interferon production, PKR activation, and cell death, all of which are dependent on MDA5 (Chung et al., 2018). Knocking down the expression of ADAR1 in astrocytes results in an upregulation of type I interferon and proinflammatory signaling pathways (McEntee et al., 2023). ADAR1 is capable of mediating nuclear A-to-I RNA editing of neuronal transcripts during the process of brain development (Behm et al., 2017). When ADAR1 is mutated, the mouse brain shows increased levels of multiple ISGS (Guo et al., 2021; Guo et al., 2022a), and white matter abnormalities with astrocytosis and microgliosis were detected at 1 year of age (Inoue et al., 2021). In the brain of mice, ADAR1 prevents the activation of MDA5 (Guo et al., 2022a; Kim et al., 2021), thereby suppressing the occurrence of inflammation and IFN response.

The existence of human ADAR1p110 is capable of suppressing age-dependent neurodegeneration in fruit fly Adar mutants (Keegan et al., 2011). When ADAR1 is deficient, it significantly affects human embryonic stem cell differentiation and neural induction (Chen et al., 2015). ADAR1 is necessary for regulating the development of two neural crest derivatives: melanocytes and Schwann cells. The specific conditional deletion of ADAR1 in the neural crest of mice results in comprehensive pigmentation and myelin sheath depletion in peripheral nerves, which are attributed to alterations in melanocyte survival and Schwann cell differentiation, respectively (Gacem et al., 2020). Furthermore, ADAR1 can interact with DDX6, a cytoplasmic RNA processing body, to jointly regulate neuronal cell differentiation (Shih et al., 2023).

ADAR1 is intricately linked to neurological diseases. Simmons M et al. first proposed that the expression of ADAR1 was significantly increased in patients with severe depression who committed suicide compared to those who did not commit suicide (Simmons et al., 2010). Intriguingly, in a separate study focusing on mice exposed to unpredictable stress (CUS), Zhang X demonstrated that the ADAR1 inducer effectively mitigated depressive-like behaviors in these mice by upregulating the BDNF protein, a biomarker associated with depression, via miR-432 (Zhang et al., 2021a). In addition, adar1 can downregulate BDNF protein and affect depressive behavior in mice by modulating circ_0000418 (Zhang et al., 2021b). Wang Y et al. confirmed in their study that ADAR1 inducers alleviate cognitive impairment by upregulating DARPP-32 protein expression in the prefrontal cortex through miR-874-3p (Wang et al., 2023). As shown in Figure 6B.

Treating rat primary cortical cell cultures with sublethal doses of glutamate (10 μM) led to a downregulation of ADAR1 protein expression, potentially indicating an adar1-mediated neuroprotective mechanism (Bonini et al., 2015). During the investigation of the neurotoxic effects of developmental sevoflurane, it was discovered that ADAR1 can suppress cell apoptosis and inflammatory responses by competing with ZBP1 for binding to Z-RNA. Furthermore, it mitigates neuronal necroptosis triggered by developmental sevoflurane through A-to-I RNA editing (Yang et al., 2024). During the treatment of nerve cells with poly-proline-arginine DPR (poly-PR), it was observed that the inhibition of ADAR1 activity mediated by poly-PR contributes to the neurotoxicity associated with amyotrophic lateral sclerosis (ALS) and frontotemporal dementia (FTD), suggesting that ADAR1 holds the potential to mitigate neurotoxicity (Suzuki and Matsuoka, 2021). When studying traumatic brain injury in humans, it was found that ADAR1 expression decreased and circHtra1 was upregulated after brain injury, promoting neuronal loss (Zheng et al., 2022). In addition, ADAR1 is a novel regulator that can promote the proliferation of activated astrocytes after ischemic stroke, reduce the production of various inflammatory cytokines after ischemic stroke, alleviate neuronal apoptosis and worsen the outcome of ischemic stroke (Cai et al., 2023b). Its normal expression also participates in regulating the neuroinflammatory cascade of Parkinson’s disease (PD) (D'Sa et al., 2025).

Intriguingly, ADAR1, apart from its aforementioned functions, also plays a role in a lesser-explored domain - memory. In the prefrontal cortex of mice, Adar1 binds to Z-DNA during fear extinction learning. Knocking down Adar1 impairs the ability to modify previously acquired fear memories, whereas the introduction of full-length Adar1 fully restores this capability (Marshall et al., 2020).

## 10 Tumor and immune escape

The multifaceted oncogenic roles of ADAR1 in tumorigenesis and immune evasion are increasingly recognized as critical drivers of cancer progression. As a key RNA-editing enzyme, ADAR1 is frequently overexpressed in a wide spectrum of malignancies, including hematological cancers and solid tumors. Its tumor-promoting functions are primarily mediated through two interconnected mechanisms: the suppression of antitumor immunity and the direct enhancement of oncogenic phenotypes. A central aspect of ADAR1’s role in immune evasion lies in its ability to dampen the innate immune response (Baker and Slack, 2022). By catalyzing widespread A-to-I editing on endogenous dsRNA, ADAR1 masks these self-molecules from being recognized as “non-self” by cytoplasmic RNA sensors such as MDA5 and RIG-I. This editing-dependent activity prevents the activation of the MAVS/IRF3 pathway, thereby blunting the production of type I interferons and the subsequent recruitment and activation of antitumor immune cells (Jia et al., 2025; Xu et al., 2025). Furthermore, ADAR1 engages in editing-independent competition with other dsRNA-binding proteins; for instance, by binding to dsRNA substrates, it physically obstructs the activation of PKR, averting PKR-mediated translational shutdown and apoptosis (Lin et al., 2023). This dual-layered immunosuppressive strategy allows cancer cells to evade immunosurveillance and create an immunologically “cold” tumor microenvironment. Beyond immune evasion, ADAR1 directly fuels tumor aggressiveness through specific RNA editing events. Hyperediting of specific transcripts can lead to gain-of-function mutations in oncogenes, such as the editing-induced recoding of *AZIN1*, which promotes cellular transformation and is linked to worse prognosis in liver and other cancers (Chen et al., 2013; Umeda et al., 2025). ADAR1 also enhances cancer cell survival, proliferation, and metastasis by editing miRNAs and their target sites, thereby dysregulating key signaling networks like the PI3K-AKT and RAS pathways (Wang Z. et al., 2025; Hata et al., 2023). The critical nature of these functions is underscored by the fact that genetic or pharmacological inhibition of ADAR1 has been shown to restore interferon signaling, reverse immune evasion, and sensitize tumors to immune checkpoint blockade therapy (Baker and Slack, 2022). Consequently, ADAR1 emerges not only as a pivotal node in cancer biology but also as a promising therapeutic target for overcoming resistance to current immunotherapies.

## 11 Summary and outlook

A common pattern of the innate immune response involves the utilization of host pattern recognition receptors (PRRs) to distinguish between foreign (non-self) and cellular (self) nucleic acids (Dorrity et al., 2023). Non-self triggers innate immune responses upon being sensed by PRRs present in all cell types (Dorrity et al., 2023), whereas self nucleic acids undergo a series of modifications to evade recognition by PRRs.However, ADAR1 lacks the ability to actively discern the origin of dsRNA.To harness the unique ability of ADAR1 in regulating innate immune responses, it is imperative to artificially and precisely manipulate its expression levels, either by knocking it down or overexpressing it.

Among all the human diseases discussed in the article, ADAR1 lacks a consistent pattern, as its presence exerts varying impacts on different diseases. However, according to current research, the existence of ADAR1 is crucial for cellular development and differentiation, and its absence can directly hinder cell differentiation or even lead to apoptosis. This prompts us to consider the impact of ADAR1 on normal cells when regulating disease treatment. It is mentioned in the latest research that ADAR1 is a broad-spectrum anti-cancer target (Wang X. et al., 2025). Clearly, ADAR1 is a crucial target worthy of exploration and utilization. Certainly, the research on ADAR1 remains incomplete, and we have yet to achieve specific and targeted modulation of ADAR1 expression levels in humans. Only by further exploring the diverse roles of ADAR1 and achieving truly precise modulation of it can we genuinely contribute to human medicine.

## Nomenclature

ADAR1: Adenosine deaminase 1
A-I: adenosine-inosine
dsRBD: dsRNA binding domain
MDA-5: melanoma differentiation antigen-5
PKR: protein kinase R
Z-RNA: left-handed RNA
A-RNA: right-handed RNA
Z-DNA: left-handed DNA
ZBP1: Z-type nucleic acid binding protein 1
RIPK3: Receptor-interacting protein kinase 3
OAS: oligoadenylate synthetase
AML: acute myeloid leukemia
p16: P16INK4a
SIRT1: Silent information regulator 1
DGCR8: DiGeorge syndrome critical region gene 8
IFN: Interferon
JAK-STAT: Janus Kinase-Signal Transducer and Activator of Transcription
SLE: systemic lupus erythematosus
RA: rheumatoid arthritis
Socs3: Suppressor of cytokine signaling 3
AGS: Aicardi-Goutières Syndrome
ISG: interferon-stimulated gene
aGVHD: acute graft-versus-host disease
dsDNA: double stranded DNA
ssRNA: sense single stranded RNA virus
NSRVs: negative single stranded RNA virus
IAV: Influenza A virus
TLR7/8: Toll-like receptors 7 and 8
HBV: Hepatitis B virus
HuR: human antigen R
CHB: chronic hepatitis B
NAFLD: Non-alcoholic fatty liver disease
HCV: Hepatitis C virus
PEG-IFN: pegylated interferon
HDV: Hepatitis D virus
VSV: Vesicular stomatitis virus
LCMV: Lymphocytic choriomeningitis virus
VACV: Cowpox virus
HIV-1: Human Immunodeficiency Virus Type 1
AIDS: acquired immunodeficiency syndrome
MeV: Measles virus
MEF: mouse embryonic fibroblasts
DENV: Dengue virus
SARS-CoV-2: Acute Respiratory Syndrome Coronavirus 2
A-to-G: Adenine to guanine
PyV: Polyoma virus
HTLV: Human T-cell lymphotropic virus
HCMV: Human Cytomegalovirus
VMC: viral myocarditis
ZIKV: Zika virus
ORFV: Orf virus
KSHV: Kaposi’s sarcoma associated herpesvirus
EBOV: Ebola virus
MARV: Marburg virus
EV-D68: Human enterovirus D68
PXR: pregnane X receptor
IRI: liver ischemia/reperfusion injury
NLRP3: NLR Family, Pyrin Domain Containing Protein 3
IPF: Idiopathic pulmonary fibrosis
PELI1: pellino homolog 1
SPRY2: Sprouty RTK signaling antagonist 2
COL3A1: collagen, type III, alpha 1
SMAD2: SMAD, mothers against DPP homolog 2
MAVS: Mitochondrial antiviral-signaling protein
NEAT1: Long non-coding RNA NEAT1
ISG: Interferon Stimulated Gene
poly-PR: poly-proline-arginine DPR
ALS: amyotrophic lateral sclerosis
FTD: frontotemporal dementia
PRRs: pattern recognition receptors
MRI: myocardial reperfusion injury
NAFLD: Nonalcoholic Fatty Liver Disease
PH: Pulmonary Hypertension
CAD: Coronary Artery Disease
TBI: Traumatic Brain Injury
PD: Parkinson’s disease
